# Sex-dimorphic growth hormone-releasing hormone (Ghrh) receptor regulation of ventromedial hypothalamic nucleus Ghrh neuron estrogen receptor variant gene expression

**DOI:** 10.1515/tnsci-2025-0373

**Published:** 2025-06-14

**Authors:** Subash Sapkota, Karen P. Briski

**Affiliations:** School of Basic Pharmaceutical and Toxicological Sciences, College of Pharmacy, University of Louisiana at Monroe, Rm 356 Bienville Building, 1800 Bienville Drive, Monroe, LA, 71201, United States

**Keywords:** growth hormone-releasing hormone receptor, ventromedial hypothalamic nucleus, insulin-induced hypoglycemia, single-cell multiplex qPCR, estrogen receptor-alpha, G protein-coupled estrogen receptor-1

## Abstract

Estradiol shapes systemic glucose homeostasis by action on ventromedial hypothalamic nucleus (VMN) targets. The neuropeptide transmitter growth hormone-releasing hormone (Ghrh) governs counterregulatory neurochemical marker mRNA expression in dorsomedial VMN (VMNdm) Ghrh/steroidogenic factor-1 (SF-1/Nr5a1) neurons. The current research used tools for *in vivo* gene silencing and single-cell laser catapult microdissection/multiplex qPCR to determine if VMN Ghrh receptor (Ghrh-R) regulates nuclear and/or membrane estrogen receptor (ER) gene transcription in those neurons. Intra-VMN Ghrh-R siRNA correspondingly up- or down-regulated baseline VMNdm Ghrh/SF-1 neuron ER-alpha (ERα) or G protein-coupled estrogen receptor-1 (GPER) transcripts in male rats; neither mRNA was affected by gene silencing in females. In each sex, hypoglycemic repression of these ER gene profiles was averted by Ghrh-R gene knockdown. Both sexes exhibited diminished baseline VMNdm Ghrh/SF-1 neuron ER-beta (ERβ) gene expression following Ghrh-R gene knockdown. ERβ mRNA was diminished (male) or unaffected (female) by hypoglycemia; Ghrh-R siRNA pretreatment enhanced transcript levels in hypoglycemic rats of either sex. Aromatase gene expression is higher in male versus female VMNdm Ghrh/SF-1 neurons and is inhibited by hypoglycemia in male rats alone. Ghrh-R gene knockdown augmented aromatase mRNA levels in each sex irrespective of glucose status. Results document glucose-dependent Ghrh-R control of VMNdm Ghrh/SF-1 neuron ERα (female), ERβ (both sexes), and GPER (both sexes) gene expression. Ongoing studies aim to characterize mechanisms that cause a hypoglycemia-associated gain of regulatory control or switch in direction (stimulatory-to-inhibitory) of control. Outcomes identify VMNdm Ghrh/SF-1 neurons as a putative neuroestradiol source in each sex and implicate Ghrh-R in hypoglycemic repression of this neurosteroid profile in males.

## Abbreviations

AROM: aromatase/CYP19A1
ERα: estrogen receptor-alpha
ERβ: estrogen receptor-beta
Ghrh: growth hormone-releasing hormone
Ghrh-R: Ghrh receptor
GPER: G protein-coupled estrogen receptor-1
IIH: insulin-induced hypoglycemia
INS: insulin
OVX: ovariectomy
*sc*
: subcutaneous
SF-1/Nr5a1: steroidogenic factor-1

## 1 Introduction

Estradiol shapes body-wide glucose homeostasis through control of carbohydrate ingestion and breakdown, liver glycogenesis and gluconeogenesis, and glucose tolerance [1,2,3,4,5]. This steroid hormone regulates secretion patterns of insulin and the counterregulatory hormones glucagon, epinephrine, and corticosterone [1,6,7,8,9]. The ventromedial hypothalamic nucleus (VMN) is a vital metabolic sensory and integrative component of the glucostatic neural network [10,11,12]. Estradiol affects glucose balance by action on VMN substrates as site-targeted exogenous hormone injection to that site changes hypoglycemic profiles in ovariectomized (OVX) female rats [13]. Documented impacts of intra-VMN estrogen receptor-alpha (ERα) or ER-beta (ERβ) receptor antagonist administration on VMN expression of protein markers for counterregulatory-constraining [γ-aminobutyric acid (GABA)] or -enhancing [glutamate (Glu); nitric oxide (NO)] neurochemicals infer that these neurotransmitters are subject to estrogenic control [14].

Progress in understanding the role of VMN in neural regulation of glucostasis will require knowledge of VMN neurons that provide input to circuitry that maintains glucose equilibrium and, moreover, characterization of metabolic, endocrine, and neurochemical stimuli that govern transmitter signaling by those cells. Dorsomedial VMN (VMNdm) growth hormone-releasing hormone (Ghrh)/steroidogenic factor-1 (SF-1/NR5A1) neurons express the counterregulation-augmenting neuropeptide transmitter Ghrh as well as genes that encode biosynthetic enzyme markers, namely glutamate decarboxylase, glutaminase, and neuronal nitric oxide synthase [15], for the characterized counterregulatory neurotransmitters γ-aminobutyric acid, glutamate, and nitric oxide, respectively. Documentation of consolidated Ghrh-mediated control of these co-expressed neurochemical surrogate gene profiles infers that this neuropeptide transmitter may manage integrated multi-modal VMNdm Ghrh/SF-1 neuron input to the glucostatic neural network. Recent studies infer that systemic glucose profiles are a crucial determinant of Ghrh neuromodulation of counterregulatory transmitter marker mRNA expression, as insulin (INS)-induced hypoglycemia (IIH) is associated with gain or loss of Ghrh control of distinctive marker gene profiles [15]. These findings infer that Ghrh signaling may mediate the effects of disturbances of glucose balance on VMNdm Ghrh nerve cell neurochemical signaling.

VMN Ghrh/SF-1 neurons are directly responsive to estradiol due to the expression of genes that encode nuclear (ERα and ERβ) and membrane (G protein-coupled estrogen receptor-1) estrogen receptors (ERs) [15]. Current research applied *in vivo* gene knockdown tools and combinative *in situ* immunocytochemistry and single-cell laser catapult microdissection/multiplex qPCR analytical methods, within the framework of an established whole-animal experimental model for IIH [16], to address the premise that VMN Ghrh receptor may impose glucose status-specific regulation of nuclear and/or membrane ER gene profiles in VMNdm Ghrh neurons. In lieu of the current U.S. National Institutes of Health policy emphasis on the investigation of sex as a critical biological variable, the present study plan incorporated adult rats of both sexes to determine if such control is implemented during eu- and/or hypoglycemia in male or female rats only or in each sex.

Brain ERs are stimulated by ligands that are taken up from the systemic circulation or are produced in local tissue [17,18]. In the brain, the androgen hormone testosterone is metabolized by aromatase enzyme-catalyzed action to neuroestradiol. Aromatase protein content and enzyme activity profiles are heterogeneous across brain structures, with the highest tissue protein levels reported for a set of forebrain loci, namely the medial preoptic area, bed nucleus of the stria terminalis, medial amygdala, and VMN [19,20,21,22,23]. Recent studies provide evidence to support the role of VMN neuroestradiol in the neural governance of glucose counterregulation [24]. Efforts to identify the VMN cell type(s) that produces neuroestradiol are at present incomplete. Current research investigated the corollary hypothesis that VMNdm Ghrh/SF-1 neurons express aromatase mRNA and that this transcript profile may be subject to glucose-dependent Ghrh-R control according to sex.

## 2 Materials and methods

### 2.1 Animals

Adult Sprague-Dawley rats were housed 2–3 animals per shoe-box cage, by sex, under a 14-h light:10-h dark cycle (lights on at 05.00 h). Rats had unrestricted access to standard laboratory chow and tap water over the duration of the study and were handled daily prior to the initiation of experimentation. Study protocols and methods were conducted in compliance with the *NIH Guide for Care and Use of Laboratory Animals*, 8th Edition, with approval by the ULM Institutional Animal Care and Use Committee.

### 2.2 Experimental design

As shown in Table 1, rats of either sex were randomly assigned on study day 1 to one out of four treatment groups (*n* = 6 male and *n* = 6 female rats/treatment group). Animals were administered ketamine/xylazine (9.0 mg ketamine/1.0 mg xylazine/0.1 mL/100 g *bw*, intraperitoneal) anesthesia by intraperitoneal injection prior to bilateral infusion (total volume: 1.0 μL; infusion rate: 3.6 μL/min) to the VMN (three-dimensional coordinates: −2.5 mm posterior to bregma, 0.6 mm lateral to midline, 9.0 mm ventral to skull surface) of control/scramble (SCR) siRNA (500 pmol; Accell Control Pool Non-Targeting; prod. no. D-001910-10-20; Horizon Discovery, Waterbeach, UK) or Ghrh-R siRNA (500 pmol; Accell rat Ghrh-R siRNA, set of 4; prod. no. EQ-089494-00-0010; Horizon Disc.) to the VMN, as described [15]. Intra-cranial siRNA delivery was achieved with a 33-G Neuros syringe (prod. no. 53496; Stoelting Co., Wood Dale, IL), guided by a Neurostar stereotactic Drill Injection Robot (Neurostar, Tubingen, Germany). In the present study plan, circulating estradiol concentrations in female subjects were made uniform by a characterized exogenous estradiol replacement protocol to minimize variability due to dynamic fluctuations in endogenous hormone secretion over the estrous cycle. While under anesthesia, female rats here were bilaterally OVX and implanted with a subcutaneous (*sc*) silastic capsule (0.062 in. *i.d*./0.125 in. *o.d*.; 10 mm/100 g *bw*) containing 30 μg 17β estradiol-3-benzoate/mL safflower oil. Average plasma estradiol concentrations achieved by this hormone replacement strategy, i.e., 22 pg/mL [25], mirror circulating steroid levels characteristic of metestrus in ovary-intact 4-day cycling adult female rats [26]. At the conclusion of surgery, rats were injected with ketophen (*sc*; Zoetis Inc., Kalamazoo, MI) and enrofloxacin (intramuscular; Bayer HealthCare LLC, Animal Health Division, Shawnee Mission, KS) and treated by topical application of 0.25% bupivacaine to closed incisions. After full recovery from anesthesia, rats were transferred to individual housing. At 09.00 h on study day 7, animals of each sex were injected *sc* with neutral protamine Hagedorn insulin (INS; 10.0 U/kg *bw*; Eli Lilly [27]) or vehicle (V; sterile diluent; Eli Lilly & Co., Indianapolis, IN). Rats were sacrificed by rapid decapitation at 1 h post-injection. Each dissected whole brain was snap-frozen by rapid immersion in liquid nitrogen-cooled isopentane and stored at −80°C.

**Table 1:** Experimental design

*Sc* Injection; Day 7	siRNA Pretreatment; Day 1	
	SCR siRNA^a^	Ghrh-R si RNA^b^
Vehicle (V)^c^	Female SCR siRNA/V; *n* = 6	Female Ghrh-R siRNA/V; *n* = 6
	Male SCR siRNA/V; *n* = 6	Male Ghrh-R siRNA/V; *n* = 6
Insulin (INS)^d^	Female SCR siRNA/INS; *n* = 6	Female Ghrh-R siRNA/INS; *n* = 6
	Male SCR siRNA/INS; *n* = 6	Male Ghrh-R siRNA/INS; *n* = 6

^a^500 pmol; Accell Control Pool Non-Targeting; prod. no. D-001910-10-20 {Horizon Discovery).

^b^500 pmol; Accell siR NA Rat Ghrh-receptor {Ghrh-R), set of 4; prod. no. EQ-099803-00-0010 {Horizon Discovery).

^c^sterile diluent; 100 uL/100 g *bw*.

^d^10.0 U neutral protamine Hagedorn insulin/kg *bw*.

### 2.3 Laser catapult microdissection of VMNdm Ghrh neurons

Consecutive fresh-frozen transverse VMN sections of 10 μm thickness were cut over a distance from −1.80 to −2.3 mm posterior to *bregma* for mounting on polyethylene naphthalate membrane-covered slides (prod. code 415190-9041-000; Carl Zeiss Microscopy LLC, White Plains, NY). Tissues were fixed with ice-cold acetone (5 min) prior to blocking with 1.5% normal goat serum (prod. code S-2000, Vector Laboratories, Burlingame, CA) in Tris-buffered saline (TBS), pH 7.4, 0.05% Triton X-100 (2 h). Sections were then incubated with a rabbit primary anti-preproGhrh antibody (prod. no. PA5-102738, 1:2,000; Invitrogen, Waltham, MA) (48–72 h; 4°C) before exposure to a horseradish peroxidase-labeled goat anti-rabbit secondary antiserum antibody (prod. no. PI-1000, 1:1,000; Vector Lab.; 1 h). Ghrh-immunoreactivity (-ir) was visualized with ImmPACT 3,3-diaminobenzidine peroxidase substrate kit reagents (prod. no. SK-4105; Vector Lab.). For each animal, individual Ghrh-ir-positive neurons were removed from tissue sections using a Zeiss P.A.L.M. UV-A microlaser IV system, as described [28,29,30,31] and placed into individual adhesive caps (prod. no. 415190-9181-000; Carl Zeiss) containing lysis buffer (4 µL; Single Shot Cell Lysis Kit, prod. no. 1725080; Bio-Rad Laboratories, Hercules, CA) for quantitative multiplex gene expression analysis.

### 2.4 Quantitative multiplex single-cell reverse-transcription PCR (RT-qPCR) analysis

#### 2.4.1 Complementary DNA (cDNA) synthesis and amplification

Single-cell lysates were centrifuged (3,000 rpm; 4°C) prior to sequential incubation in an iCyclerQ RT-PCR Detection System (Bio-Rad) at 25°C (10 min) and 75°C (5 min). Sample RNA quantity and purity were analyzed by NanoDrop spectrophotometry (prod. code ND-ONE-W, ThermoFisherScientific, Waltham, MA). Reverse-transcription of single-cell mRNA samples to cDNA was achieved by adding cDNA synthesis buffer (1.5 µL; iScript™ Advanced cDNA Synthesis Kit.; prod. code 1725038; Bio-Rad) before initial incubation at 46°C (20 min) and then at 95°C (1 min), as described [32,33,34]. PrimePCR™ PreAmp for SYBR^®^ Green Assays for ESR1/ERalpha (ERα) (prod. code qRnoCID0009588), ESR2/ERbeta (ERβ) (prod. code qRnoCID0008785), G protein-coupled estrogen receptor-1 (GPER) (prod. code qRnoCED0007818), aromatase/CYP19A1 (prod. code qRnoCID0007793), Ghrh (prod. code qRnoCID0007723), and GAPDH (prod. code qRnoCID0057018; Bio-Rad) were combined with SsoAdvanced™ PreAmp Supermix (prod. code 1725160; Bio-Rad) to prepare a pre-amplification master mix, which was added (9.5 µL) to each cDNA sample prior to thermal cycler incubation at an initial temperature of 95°C (3 min), followed by 18 cycles of incubation of sequential incubation at 95°C (15 s)/58°C (4 min). RT-qPCR analysis: IDTE (prod. code 11-05-01-05; 1× TE solution; Integrated DNA Technologies, Inc., Coralville, IA)-diluted pre-amplified cDNA samples (2 µL) were combined with Bio-Rad primers (ESR1 [0.5 µL; prod. code qRnoCID0009588], ESR2 [0.5 µL; prod. code qRnoCID0008785], GPER [0.5 µL; prod. code qRnoCED0007818], aromatase/CYP19A1 [0.5 µL; prod. code. qRnoCID0007793], Ghrh [0.5 µL; prod. code qRnoCID0007723], and GAPDH [0.5 µL; prod. code qRnoCID0057018]) and iTaq™ Universal SYBR^®^ Green Supermix (5 µL; prod. code 1725121; Bio-Rad). PCR samples were added to individual wells of 384-well hard-shell PCR plates (prod. code HSP3805, Bio-Rad) for analysis in a CFX384™ Touch Real-Time PCR Detection System (Bio-Rad) as follows: initial 95°C denaturation (30 s), followed by 40 cycles of (1) incubation at 95°C (3 s) and (2) 30 s incubation at 60°C for ESR1; 59.9°C for ESR2; 59.8°C for GPER; 59.6°C for aromatase/CYP19A1, 58.5°C for Ghrh, or 57.3°C for GAPDH, respectively. Melt curve analyses were carried out to identify nonspecific products and primer dimers. Data were normalized by the comparative Ct (2^−ΔΔCt^) method [35].

### 2.5 Laser-microdissected Ghrh-ir neuron Ghrh-R protein expression: Western blot analysis

Triplicate independent cell lysate pools (*n* = 50 Ghrh-ir neurons/pool/treatment group) were created for each treatment group for Ghrh-R protein measurement. Sample pool proteins were subjected to electrophoresis separation in Bio-Rad TGX 10% stain-free gels (prod. code 1610183; Bio-Rad). Total in-gel protein was measured by Stain Free imaging technology as a loading control [14,30,36]. This state-of-the-art Western blot normalization method significantly reduces data variability through augmented measurement accuracy and precision [37,38]. After electrophoresis, gels were UV light-activated (1 min) in a Bio-Rad ChemiDoc MP Imaging System to quantify the total protein separated in each gel lane. Proteins were transferred to 0.45-μm PVDF-Plus membranes (prod. code 121639; Data Support Co., Panorama City, CA), for automated wash and antibody incubation processing by FreedomRocker™ Blotbot^®^ (Next Advance, Inc., Troy, NY). Non-specific immunoreagent binding to membranes was abated by blocking with Tris-buffer saline, pH 7.4, 10 mM tris hydrochloride, and 50 mM sodium chloride (TBS) supplemented with 0.1% Tween-20 and 2% bovine serum albumin. Membranes were then incubated with a rabbit anti-Ghrh-R primary polyclonal antiserum (prod. code PA5-121195; 1:1,000; Invitrogen) (36–42 h; 4°C) [15], prior to sequential exposure to goat anti-rabbit horseradish peroxidase-labeled secondary antibodies (1:5,000; prod. code NEF812001EA; PerkinElmer, Waltham, MA) and SuperSignal West Femto chemiluminescent substrate (prod. code 34096; ThermoFisherSci.). Bio-Rad Stain Free gel chemistry features direct incorporation of a proprietary trihalo compound that lacks inherent fluorescence but renders all in-gel proteins fluorescent (and thus measurable by optical density) upon UV photoactivation. Chemiluminescence OD values obtained for target protein bands were normalized to the sum of in-lane protein OD by ChemiDoc MP Image Lab™ 6.0.0 software. The *Y*-axis of data figures presented here depicts mean normalized OD measures. Each Western blot analysis employed precision plus protein molecular weight dual color standards (prod. no. 161-0374, Bio-Rad).

### 2.6 Statistics

Mean normalized VMNdm neuron mRNA measures were analyzed among treatment groups by three-way analysis of variance and Student–Newman–Keuls *post hoc* test. Differences of *p* < 0.05 were considered significant. In each figure, statistical differences between specific pairs of treatment groups are denoted as follows: *p < 0.05; **p < 0.01; ***p < 0.001.


**Ethical approval:** The research related to animals’ use has been complied with all the relevant national regulations and institutional policies for the care and use of animals. All animal experimental procedures were performed in accordance with the *National Institutes of Health Guide for the Care and Use of Laboratory Animals*, 8th Edition, under the approval of the University of Louisiana at Monroe IACUC committee, as stated in the submitted manuscript. The sex of the animals used is included, along with a discussion of sex impacts on study outcomes.

## 3 Results

VMNdm Ghrh/SF-1 neurons are a potential target for estradiol control of counterregulation. Ghrh-R control of counterregulatory neurochemical surrogate gene expression in these cells and systemic counterregulatory hormone secretion varies between conditions of glucose sufficiency (euglycemia) versus deficiency (hypoglycemia). The present experimental design involved site-targeted bilateral Ghrh-R siRNA administration to the male or female rat VMN in advance of vehicle (V) or INS injection. Single-cell laser catapult microdissection/multiplex qPCR analytical tools were used to address the novel premise that VMN Ghrh-R may impose glucose-contingent regulation of VMNdm Ghrh neuron ER variant RNA profiles in one or both sexes.

**Figure 1:**
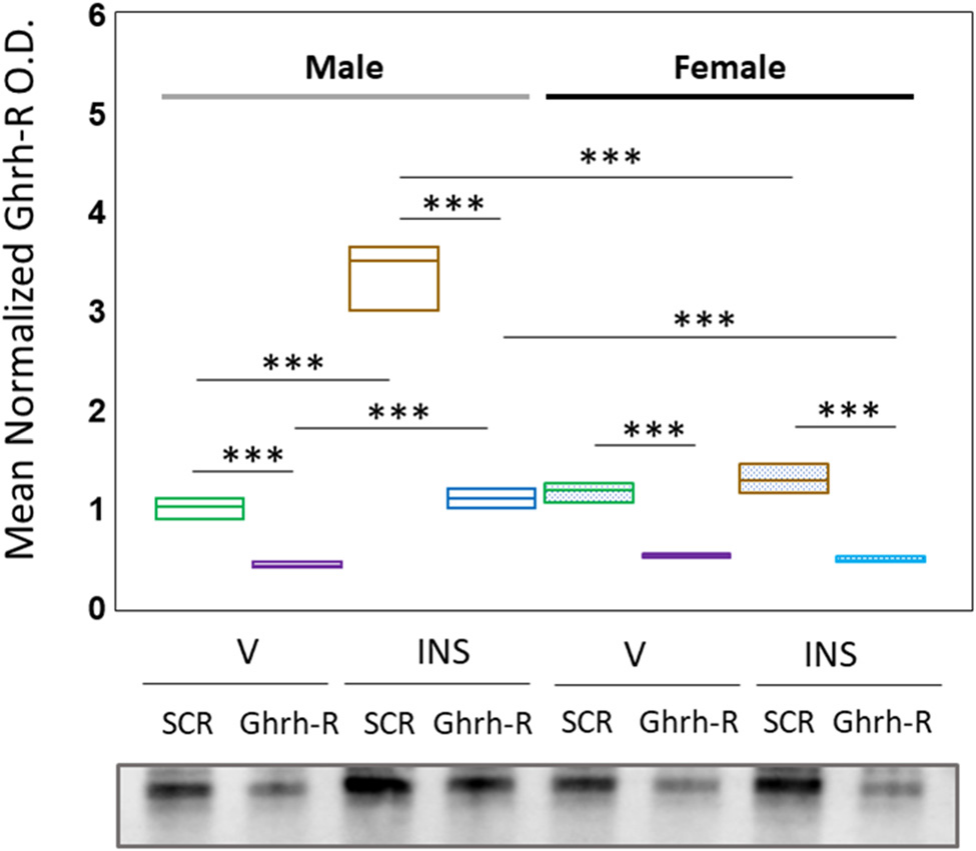
Effects of VMN growth hormone-releasing hormone receptor (Ghrh-R), gene knockdown on male or female dorsomedial VMN (VMNdm), and Ghrh-immunopositive nerve cell Ghrh-R protein expression. Groups of male and female rats (*n* = 6 males and *n* = 6 females per group) were pretreated by bilateral intra-VMN scramble (SCR) or Ghrh-R siRNA administration 7 days before subcutaneous (sc) injection of vehicle (V) or neutral protamine Hagedorn insulin (INS; 10.0 U/kg *bw*). Brain tissue was harvested by dissection 1 h after injections. Individual Ghrh-immunopositive neurons were laser catapult-microdissected from 10 μm-thick fresh frozen sections cut through the VMNdm. For each treatment group, aliquots of VMNdm cell lysate obtained from individual subjects were combined to create triplicate sample pools for Western blot analysis of Ghrh-R protein. Data are presented here in box-and-whisker plot format, which displays the median, lower and upper quartiles, and lower and upper extremes of a data set. Data illustrate normalized Ghrh-R protein optical density (OD) values for male (*at left*) and female (*at right*) rat treatment groups: SCR siRNA/V (green box-and-whisker plots, male: no fill, *n* = 3; female: stippled fill, *n* = 3); Ghrh-R siRNA/V (purple box-and-whisker plots; male: no fill, *n* = 3; female: stippled fill, *n* = 3); SCR siRNA/INS (brown box-and-whisker plots; male: no fill, *n* = 3; female: stippled fill, *n* = 3); and Ghrh-R siRNA/INS (blue box-and-whisker plots; male: no fill, *n* = 3; female: stippled fill, *n* = 3). Mean OD data were analyzed among treatment groups by three-way analysis of variance and Student–Newman–Keuls *post hoc* test. Statistical differences between discrete pairs of treatment groups are denoted as follows: *p < 0.05; **p < 0.01; ***p < 0.001.

Figure 1 depicts patterns of VMNdm Ghrh/SF-1 neuron Ghrh-R protein expression in Ghrh-R siRNA-pretreated V- or INS-injected in male versus female rat [*F*_(7,88)_ = 129.30, *p* < 0.001; sex main effect: *F*_(1,88)_ = 105.65, *p* < 0.001; pretreatment main effect: *F*_(1,88)_ = 331.27, *p* < 0.001; treatment main effect: *F*_(1,88)_ = 174.78, *p* < 0.001; sex/pretreatment interaction: *F*_(1,88)_ = 34.47, *p* < 0.001; sex/treatment interaction: *F*_(1,88)_ = 155.52, *p* < 0.001; pretreatment/treatment interaction: *F*_(1,88)_ = 61.91, *p* < 0.001; and sex/pretreatment/treatment interaction: *F*_(1,88)_ = 41.51, *p* < 0.001. Data indicate that Ghrh-R siRNA significantly decreased Ghrh-R protein levels in eu- and hypoglycemic rats of each sex. Outcomes confirm the efficacy of the gene knockdown paradigm used here for down-regulation of Ghrh-R protein expression in this neuron population.

**Figure 2:**
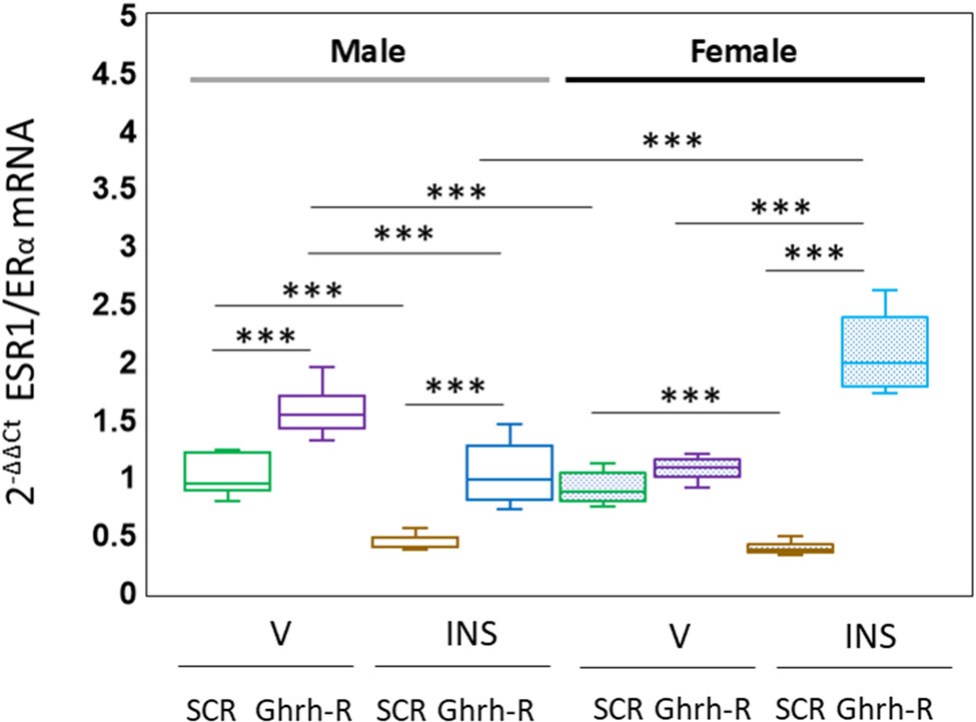
Effects of VMN Ghrh-R gene knockdown on estrogen receptor-alpha (ESR1/ERα), gene expression in male or female VMNdm Ghrh-immunopositive neurons. Individual laser catapult-microdissected VMNdm Ghrh-immunopositive neurons were collected from each animal for multiplex single-cell qPCR analysis. mRNA data were normalized to the housekeeping gene GAPDH by the 2^−ΔΔCt^ method. Data are presented here in box-and-whisker plot format, which displays the median, lower and upper quartiles, and lower and upper extremes of a data set. Plots depict normalized ESR1/ERα mRNA measures for male (*at left*) and female (*at right*) rat treatment groups. Treatment groups are identified as follows: SCR siRNA/V (green box-and-whisker plots, male: no fill, *n* = 12; female: stippled fill, *n* = 12); Ghrh-R siRNA/V (purple box-and-whisker plots; male: no fill, *n* = 12; female: stippled fill, *n* = 12); SCR siRNA/INS (brown box-and-whisker plots; male: no fill, *n* = 12; female: stippled fill, *n* = 12); Ghrh-R siRNA/INS (blue box-and-whisker plots; male: no fill, *n* = 12; female: stippled fill, *n* = 12). Gene transcript data were analyzed by three-way analysis of variance and Student–Newman–Keuls *post hoc* test. Statistical differences between discrete pairs of treatment groups are denoted as follows: *p < 0.05; **p < 0.01; ***p < 0.001.

Data in Figure 2 illustrate VMN Ghrh-R gene silencing effects on VMNdm Ghrh/SF-1 neuron ESR1/ERα gene expression in vehicle (V) or insulin (INS)-injected male (*at left*; no-fill box-and-whisker plots) and female (*at right*; stipple-filled box-and-whisker plots) rats. Outcomes of data statistical analysis are as follows: *F*_(7,88)_ = 118.83, *p* < 0.001; sex main effect: *F*_(1,88)_ = 6.83, *p* = 0.011; pretreatment main effect: *F*_(1,88)_ = 441.20, *p* < 0.001; treatment main effect: *F*_(1,88)_ = 21.05, *p* < 0.001; sex/pretreatment interaction: *F*_(1,88)_ = 22.33, *p* < 0.001; sex/treatment interaction: *F*_(1,88)_ = 122.53, *p* < 0.001; pretreatment/treatment interaction: *F*_(1,88)_ = 115.02, *p* < 0.001; and sex/pretreatment/treatment interaction: *F*_(1,88)_ = 102.84, *p* < 0.001. Data show that Ghrh gene silencing augmented ERα mRNA profiles in male, but not in female rat VMNdm Ghrh/SF-1 neurons (Ghrh-R siRNA/V [unfilled purple box-and-whisker plot] versus SCR siRNA/V [unfilled green box-and-whisker plot]). Hypoglycemia decreased this gene profile in each sex (SCR siRNA/INS [brown box-and-whisker plots; unfilled: male, stippled-filled, female] versus SCR siRNA/V [green box-and-whisker plots; unfilled: male, stipple-filled, female]). Ghrh-R siRNA pretreatment averted this inhibitory transcriptional response in male and female rats (Ghrh-R siRNA/INS [blue box-and-whisker plots; unfilled: male, stippled-filled, female] versus SCR siRNA/INS [brown box-and-whisker plots; unfilled: male, stipple-filled, female]).

**Figure 3:**
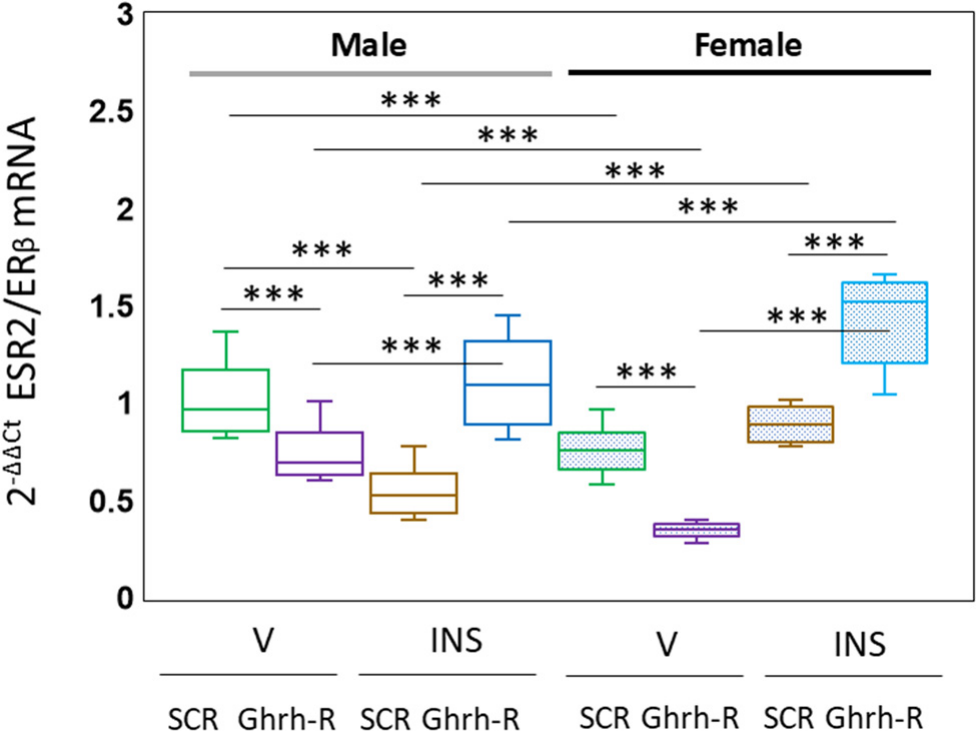
Ghrh-R-dependent VMNdm Ghrh/SF-1 neuron ER-beta (ESR2/ERβ) mRNA profiles in vehicle- or insulin-injected male and female rats. Results present normalized ESR2/ERβ mRNA measures for male (*at left*) or female (*at right*) groups of rats treated as follows: SCR siRNA/V (green box-and-whisker plots; male: no fill, *n* = 12; female: stippled fill, *n* = 12); Ghrh-R siRNA/V (purple box-and-whisker plots; male: no fill, *n* = 12; female; stipple fill, *n* = 12); SCR siRNA/INS (brown box-and-whisker plots; male: no fill, *n* = 12; female: stippled fill, *n* = 12); and Ghrh-R siRNA/INS (blue box-and-whisker plots; male: no fill, *n* = 12; female: stippled fill, *n* = 12). Normalized mRNA data were analyzed by three-way analysis of variance and Student–Newman–Keuls *post hoc* test. Statistical differences between discrete pairs of treatment groups are denoted as follows: *p < 0.05; **p < 0.01; ***p < 0.001.

Data presented in Figure 3 illustrate patterns of VMNdm Ghrh/SF-1 neuron ESR2/ERβ mRNA expression in eu- or hypoglycemic male and female rats after VMN Ghrh-R siRNA administration. Statistical analysis of data depicted in Figure 2 showed the following results: *F*_(7,88)_ = 60.05, *p* < 0.001; sex main effect: *F*_(1,88)_ = 0.01, *p* = 0.908; pretreatment main effect: *F*_(1,88)_ = 11.49, *p* = 0.001; treatment main effect: *F*_(1,88)_ = 82.72, *p* < 0.001; sex/pretreatment interaction: *F*_(1,88)_ = 1.62, *p* = 0.207; sex/treatment interaction: *F*_(1,88)_ = 114.08, *p* < 0.001; pretreatment/treatment interaction: *F*_(1,88)_ = 209.55, *p* < 0.001; sex/pretreatment/treatment interaction: *F*_(1,88)_ = 0.85, *p* = 0.359. Outcomes show that VMN Ghrh-R siRNA administration diminishes baseline ERβ gene expression in euglycemic male and female rats, yet this gene knockdown paradigm increases this mRNA profile in hypoglycemic animals of each sex.

**Figure 4:**
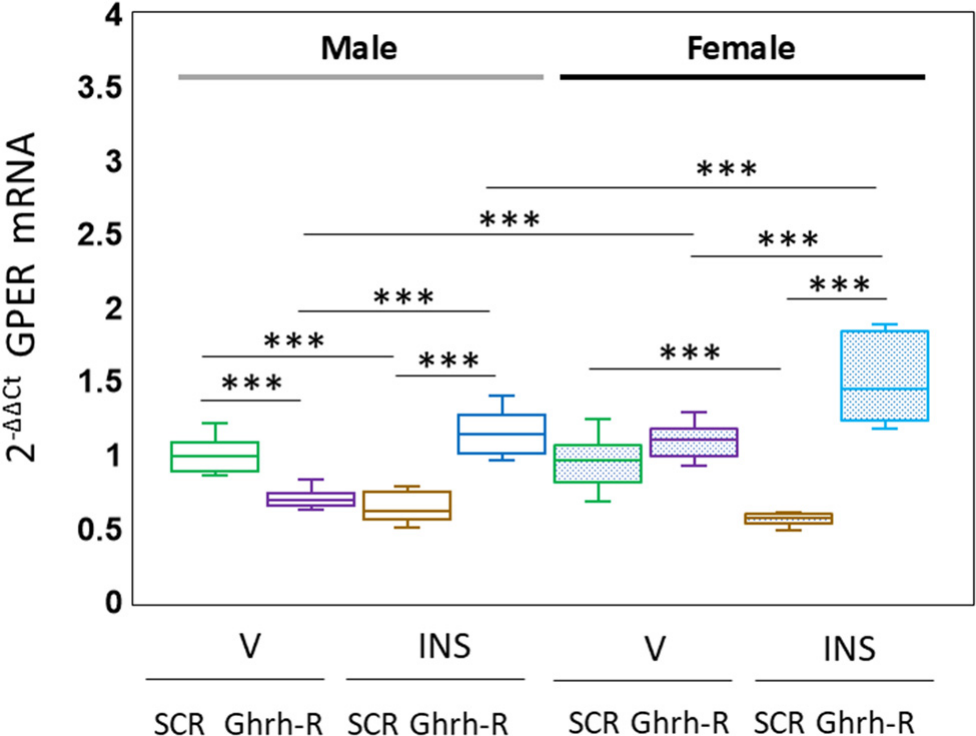
Effects of VMN Ghrh gene knockdown on G protein-coupled estrogen receptor-1 (GPER) gene expression in male or female rat VMNdm Ghrh neurons. Data for normalized GPER mRNA measures for male (*at left*) or female (*at right*) treatment groups are depicted here in box-and-whisker plot format. Treatment groups are represented as follows: SCR siRNA/V (green box-and-whisker plots; male: no fill, *n* = 12; female: stippled fill, *n* = 12); Ghrh-R siRNA/V (purple box-and-whisker plots; male: no fill, *n* = 12; female; stipple fill, *n* = 12); SCR siRNA/INS (brown box-and-whisker plots; male: no fill, *n* = 12; female: stippled fill, *n* = 12); and Ghrh-R siRNA/INS (blue box-and-whisker plots; male: no fill, *n* = 12; female: stippled fill, *n* = 12). Normalized mRNA data were analyzed by three-way analysis of variance and Student–Newman–Keuls *post hoc* test. Statistical differences between discrete pairs of treatment groups are denoted as follows: *p < 0.05; **p < 0.01; ***p < 0.001.

Results shown in Figure 4 depict VMNdm Ghrh/SF-1 nerve cell GPER mRNA profiles in Ghrh-R siRNA – pretreated male versus female rats. Outcomes of data statistical analysis are as follows: *F*_(7,88)_ = 52.40, *p* < 0.001; sex main effect: *F*_(1,88)_ = 27.55, *p* < 0.001; pretreatment main effect: *F*_(1,88)_ = 107.97, *p* < 0.001; treatment main effect: *F*_(1,88)_ = 1.22, *p* = 0.273; sex/pretreatment interaction: *F*_(1,88)_ = 53.54, *p* < 0.001; sex/treatment interaction: *F*_(1,88)_ = 0.17, *p* = 0.681; pretreatment/treatment interaction: *F*_(1,88)_ = 176.35, *p* < 0.001; and sex/pretreatment/treatment interaction: *F*_(1,88)_ = 0.02, *p* = 0.888. Data indicate that Ghrh-R gene knockdown reduced VMNdm Ghrh/SF-1 nerve cell GPER gene transcription in V-injected male rats but did not alter this mRNA profile in females. In both sexes, hypoglycemic suppression of GPER gene transcripts was reversed by Ghrh-R siRNA pretreatment.

**Figure 5:**
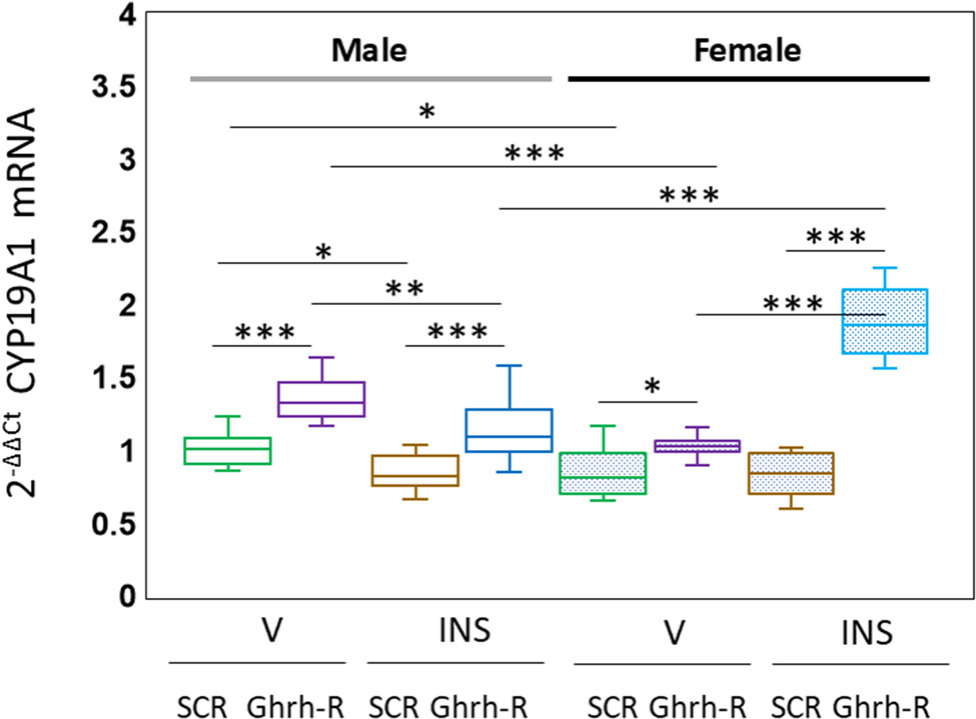
Ghrh-R regulation of VMNdm Ghrh/SF-1 neuron aromatase gene expression in eu- or hypoglycemic male and female rats. Results present normalized aromatase mRNA values male (*at left*) or female (*at right*) groups of rats treated as follows: SCR siRNA/V (green box-and-whisker plots; male: no fill, *n* = 12; female: stippled fill, *n* = 12); Ghrh-R siRNA/V (purple box-and-whisker plots; male: no fill, *n* = 12; female; stipple fill, *n* = 12); SCR siRNA/INS (brown box-and-whisker plots; male: no fill, *n* = 12; female: stippled fill, *n* = 12); and Ghrh-R siRNA/INS (blue box-and-whisker plots; male: no fill, *n* = 12; female: stippled fill, *n* = 12). Normalized mRNA data were analyzed by three-way analysis of variance and Student–Newman–Keuls *post hoc* test. Statistical differences between discrete pairs of treatment groups are denoted as follows: *p < 0.05; **p < 0.01; ***p < 0.001.

Figure 5 depicts the effects of VMN Ghrh-R gene silencing on VMNdm Ghrh/SF-1 neuron aromatase gene expression in vehicle (V) or insulin (INS)-injected male (*at left*; no-fill box-and-whisker plots) and female (*at right*; stipple-filled box-and-whisker plots) rats. Outcomes of data statistical analysis are as follows: *F*_(7,88)_ = 60.86, *p* < 0.001; sex main effect: *F*_(1,88)_ = 3.29, *p* = 0.073; pretreatment main effect: *F*_(1,88)_ = 210.69, *p* < 0.001; treatment main effect: *F*_(1,88)_ = 13.75, *p* < 0.001; sex/pretreatment interaction: *F*_(1,88)_ = 20.50, *p* < 0.001; sex/treatment interaction: *F*_(1,88)_ = 90.96, *p* < 0.001; pretreatment/treatment interaction: *F*_(1,88)_ = 38.22, *p* < 0.001; and sex/pretreatment/treatment interaction: *F*_(1,88)_ = 48.62, *p* < 0.001. Data show that baseline aromatase gene expression in this nerve cell population is significantly up-regulated in both male and female rats in response to Ghrh-R gene knockdown. Hypoglycemia inhibited this gene profile in male, but not in female rats. Pretreatment with Ghrh-R siRNA elevated aromatase mRNA levels in hypoglycemic rats of each sex.

**Figure 6:**
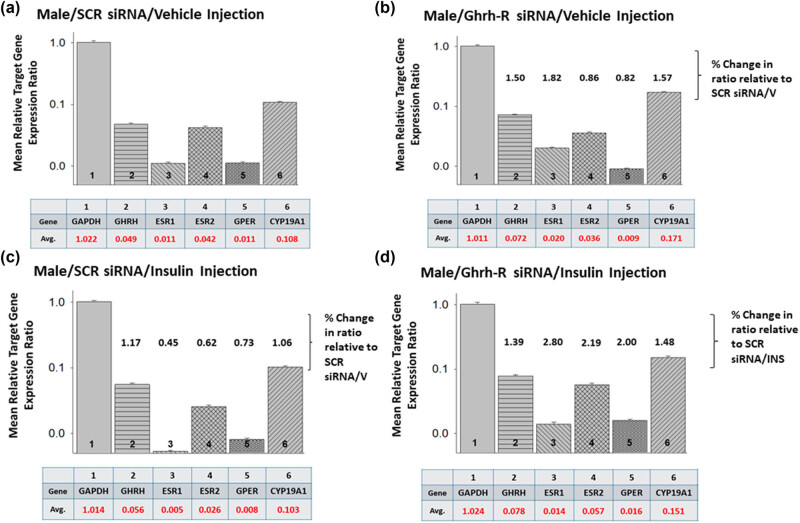
Mean relative expression ratios of ER variant and aromatase mRNAs analyzed for eu- versus hypoglycemic male rat VMNdm Ghrh/SF-1 neurons. Multiplex single-cell qPCR data acquired from laser catapult-microdissected Ghrh-ir neurons were used to establish mean ratios for target gene expression levels relative to GAPDH mRNA. Averaged mean ratio values were derived from *n* = 12 laser catapult-microdissected VMNdm Ghrh-ir neurons from male rats treated as follows: SCR siRNA/V (a), Ghrh-R siRNA/V (b), SCR siRNA/INS (c), and Ghrh-R siRNA/INS (d). In each figure panel, mean relative gene expression data are depicted in graphical (*top*; bars with S.E.M.; note the exponential *Y*-axis scale) and tabular (*bottom*); (1) top row: gene name and (2) bottom row: average expression ratio value) formats. (b and c) A numerical notation above individual relative expression ratio bars to denote percentage change versus the corresponding mean target gene/Ghrh mRNA ratio for SCR siRNA/V control group. Notations associated with mean ratio bars in (d) indicate percentage change in proportionate target gene expression compared to corresponding ratios for the SCR siRNA/INS treatment group.

Figure 6 presents expression ratios of ER variant and aromatase gene mRNAs for VMNdm Ghrh/SF-1 neurons harvested from SCR or Ghrh-R siRNA-pretreated *sc* V- (Figure 6a and b) or INS- (Figure 6c and d) injected male rats. In each panel, average ratios of individual target gene versus GAPDH mRNA profiles, identified by number, are depicted in graphical (note the exponential *Y*-axis scale) and tabular formats. Figure S1 depicts Ghrh nerve cell GAPDH gene expression across male and female rat treatment groups. Data in Figure 6a show that under euglycemic conditions, proportionate ER gene expression varies as indicated by differential average values, namely higher relative levels of ERβ versus other receptor subtypes. Figure 6b illustrates Ghrh-R knockdown effects on individual gene expression ratios and provides numerical notation of change in ratio values versus SCR siRNA/V controls. Data show that this treatment increases or decreases proportionate ERα or ERβ gene transcription, respectively, inferring that Ghrh-R may inhibit or stimulate the relative expression of these genes. Knockdown-associated up-regulation of aromatase mRNA points to evident the suppression of proportionate expression of this gene by Ghrh-R signaling. Comparison of VMNdm Ghrh/SF-1 neuron relative target gene expression in hypo- (Figure 6c) versus euglycemic (Figure 6c) males infers that in this sex, glucose imbalance may suppress the relative expression of nuclear ER variant genes in the absence of change in aromatase transcription. Notably, Ghrh-R siRNA reverses hypoglycemia-associated down-regulation of proportionate ERα and ERβ gene expression, indicative of a primary role for this neurochemical signal in these inhibitory responses. Comparison with the Ghrh-R siRNA/V group suggests that during hypoglycemia, Ghrh-R may gain control of GPER transcription.

**Figure 7:**
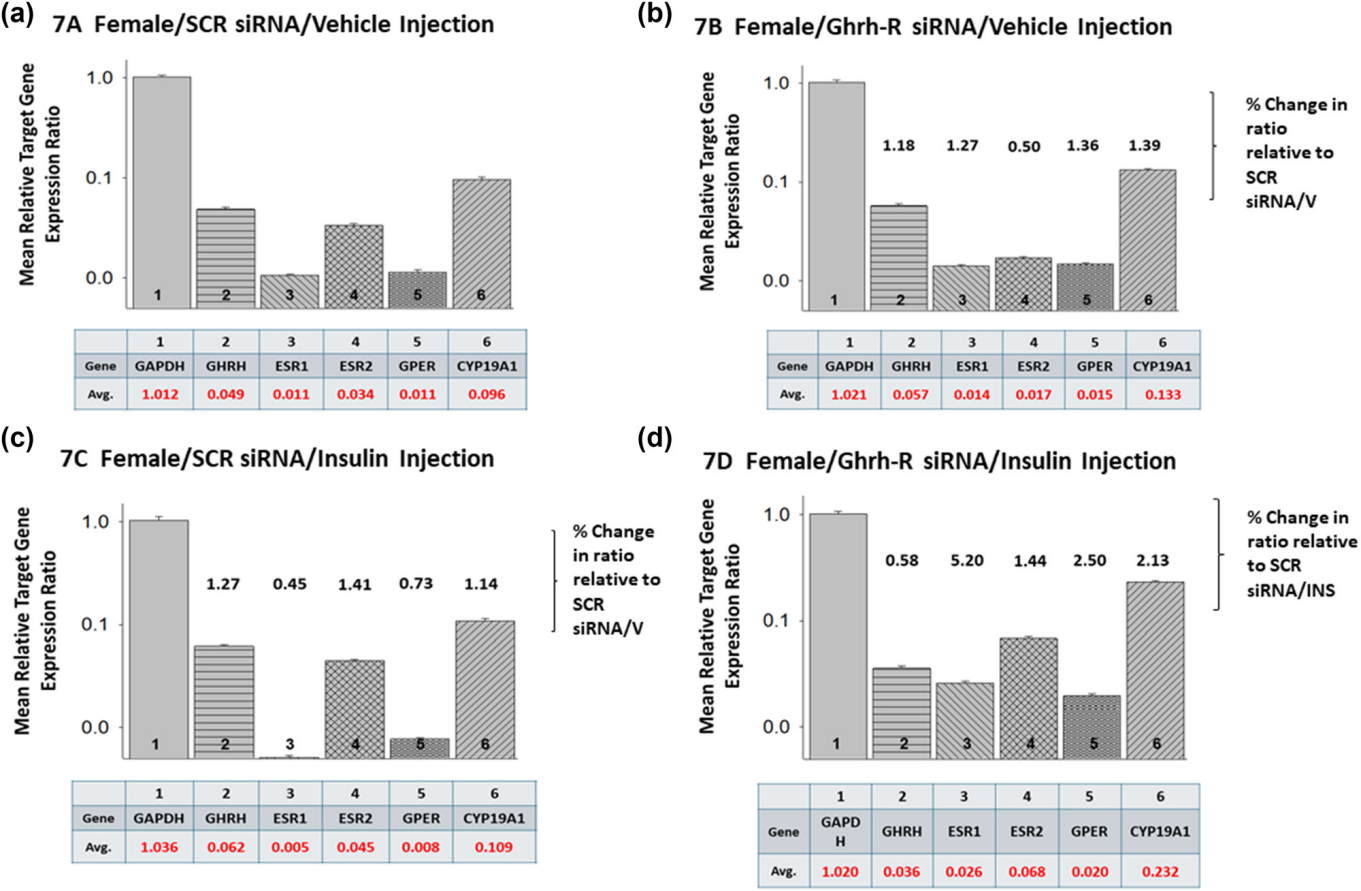
Relative VMNdm Ghrh/SF-1 neuron target gene expression ratios for SCR versus Ghrh-R siRNA-pretreated, V- or INS-injected female rats. Mean ratios for Ghrh/SF-1 nerve cell target gene expression relative to GAPDH mRNA were constructed using multiplex single-cell qPCR data derived from individual laser catapult-microdissected Ghrh-ir neurons harvested from SCR siRNA/V (a), Ghrh-R siRNA/V (b), SCR siRNA/INS (c), or Ghrh-R siRNA/INS (d) groups of female rats. Mean relative gene expression values for each treatment group are presented in a bar graph (*at top*) and accompanying table (*at bottom*). (b and c) A numerical notation above individual relative expression ratio bars to indicate percentage change versus the corresponding target gene/Ghrh mRNA ratio for SCR siRNA/V control group. Notations associated with mean ratio bars in (d) indicate percentage change in proportionate target gene expression, compared to corresponding ratios for the SCR siRNA/INS treatment group.

Data presented in Figure 7 illustrate target gene versus Ghrh mRNA ratios in VMNdm Ghrh/SF-1 neurons from SCR siRNA/V (Figure 7a), Ghrh-R siRNA/V (Figure 7b), SCR siRNA/INS (Figure 7c), and Ghrh-R siRNA/INS (Figure 7d) female rats. Like the male rats, euglycemic female rats exhibit divergent target gene expression relative to GAPDH (Figure 7a). In the female, Ghrh-R gene silencing had converse effects on proportionate aromatase and ERβ gene transcription, inferring that Ghrh-R signaling may down- or up-regulate relative expression of these genes (Figure 7b). Hypoglycemic female rats showed a decline or augmentation of relative ERα or ERβ transcription, respectively, with a change in aromatase gene profiles (Figure 7c). Data in Figure 7d infer that in this sex, during hypoglycemia, Ghrh-R imposes a greater inhibitory tone on relative ERα, ERβ, and GPER gene transcription compared to the Ghrh-R siRNA/V group.

## 4 Discussion

Ghrh-R governs counterregulatory neurotransmission by VMNdm Ghrh/SF-1 neurons. Present studies analyzed ER mRNA expression in these cells by single-cell multiplex qPCR methods to address the premise that in one or both sexes, Ghrh-R may regulate cellular receptivity to ER variant signaling according to systemic glucose status. Outcomes show that in euglycemic rats, Ghrh-R imposes sex-specific control of baseline ER variant gene expression in VMNdm Ghrh/SF-1 nerve cells, as Ghrh-R gene knockdown altered all nuclear and cytoplasmic ER mRNA profiles in males but affected only ERβ transcription in the female. Data disclose sex-contingent ER variant transcriptional reactivity to IIH; all ER variant gene profiles were down-regulated in INS-injected male rats yet only ERα and GPER mRNA levels declined in hypoglycemic females. Results disclose hypoglycemic control of Ghrh-R regulation of distinctive ER variant mRNA levels in each sex, as males show a shift in direction of control (i.e., positive-to-negative) of VMNdm Ghrh/SF-1 neuron ERβ and GPER gene expression in male while females exhibit a gain of Ghrh-R regulation of ERα and GPER transcription alongside a switch from stimulatory-to-inhibitory control of ERβ mRNA. Current results provide unique evidence that VMNdm Ghrh/SF-1 neurons express Ghrh-R-sensitive aromatase mRNA and that IIH causes sex-dimorphic adjustments in this gene profile. VMNdm Ghrh/SF-1 neurons emerge here as a potential central target for estradiol management of glucose counterregulation. Study outcomes support the notion that Ghrh-R may shape Ghrh/SF-1 nerve cell counterregulatory neurochemical transmission according to sex and systemic glucose status, in part, through control of ER variant-specific estradiol input to these cells.

Considering that VMNdm Ghrh/SF-1 neurons express Ghrh-R mRNA [15], observed Ghrh-R gene silencing effects on ER gene expression likely reflect, in part, diminution of direct Ghrh input to these cells. Alternatively, the prospect that Ghrh may also indirectly affect Ghrh/SF-1 nerve cell ER gene transcription, under eu and/or hypoglycemic conditions, via control of afferent neural input to those cells should not be discounted. A current knowledge gap concerns the post-receptor signaling mechanism(s) that conveys, in each sex, Ghrh-R regulation of VMNdm Ghrh/SF-1 neuron ER variant gene transcription during glucose balance versus imbalance. VMNdm Ghrh/SF-1 neurons evidently engage in metabolic self-monitoring as these cells express mRNAs that encode characterized metabolic sensory bio-machinery, i.e. the glucose sensor glucokinase and energy sensor 5′-AMP-activated protein kinase catalytic subunit isoforms [39]. Further research is warranted to examine whether, in one or both sexes, hypoglycemia-associated changes in VMNdm Ghrh/SF-1 neuron glucose uptake and metabolism and/or net energy balance influence the volume or direction of activity of Ghrh-R-sensitive signal transduction pathways that govern ER gene expression.

Single-cell qPCR data reported here disclose sex-contingent Ghrh-R control of baseline VMN Ghrh/SF-1 nerve cell ERα and GPER gene transcription, as both mRNA profiles were up-regulated (male) or unaffected by (female) receptor siRNA administration. These outcomes infer that receptor input may diminish the expression of these distinctive nuclear and cytoplasmic ERs in males alone. This presumption should be considered speculative until analytical methods of requisite sensitivity for protein quantification in single brain cell samples become available. Aside from the above-stated need to elucidate the signal transduction mechanism(s) that mediate Ghrh-R effects on VMNdm Ghrh/SF-1 ERα and GPER gene transcription, there is a critical need to understand if and how sex-specific regulation of post-receptor signaling is achieved within the context of normoglycemia. An unexpected result reported here involves the characterization of Ghrh-R inhibition of ERα and GPER ER transcripts in hypoglycemic male rats, an action that reflects a switch from positive-to-negative receptor control of the latter mRNA. Present studies did not answer the important question of the molecular mechanism(s) that mediates glucose-dependent adjustment in receptor influence on GPER gene transcription. Meanwhile, in the female, data document a hypoglycemia-associated gain in inhibitory Ghrh-R control of VMNdm Ghrh/SF-1 neuron ERα and GPER gene profiles. Thus, in the latter sex, it will be imperative to identify intra- versus extracellular cues of metabolic imbalance that may allow Ghrh-R signaling to affect transcription of those ER mRNAs only during glucose insufficiency. Ghrh-R signaling is evidently a positive stimulus for baseline VMNdm Ghrh/SF-1 nerve cell ERβ gene expression in each sex, yet this receptor paradoxically inhibits this gene profile in hypoglycemic male and female rats. As ERβ mRNA levels did not vary here between V- versus INS-injected female rats, suppressive effects of this receptor on gene transcription are obviously counter-balanced in that sex by as-yet-unknown positive stimuli. Further work will be required to elucidate the molecular mechanism(s) that operates in each sex to enact a shift in the Ghrh-R direction of control of VMNdm Ghrh/SF-1 neuron ERβ gene transcription in response to hypoglycemia. The possibility that metabolic cues may regulate ER variant gene/gene product expression in these neurons by Ghrh-R-independent as well as -dependent mechanisms also merits investigation. Study outcomes underscore the need to characterize the distinctive role of individual VMN Ghrh/SF-1 nerve cell ER variants on eu- and hypoglycemic patterns of counterregulatory neurotransmission by these neurons and on peripheral counterregulatory hormone secretory patterns.

Simultaneous acquisition of individual gene expression profiles by multiplex qPCR analysis creates an option for evaluation of the relative expression of multiple target genes within a common cellular source. Here, a compilation of relative expression ratios for ERα, ERβ, and GPER shows that for euglycemic rats of each sex, ERβ transcription predominates in VMNdm Ghrh/SF-1 neurons compared to other ER variant genes. Ghrh-R gene knockdown likely affects cellular processing of estradiol signals considering ensuing adjustments in proportionate ER variant gene expression, including reductions in ERβ mRNA profiles. Interestingly, on the one hand, hypoglycemia evidently diminishes receptivity of these neurons to estradiol in the male as relative expression of all three ER genes was decreased, yet did not affect proportionate ERβ expression in the female. Glucose status evidently determines the magnitude of Ghrh-R regulation of relative ER variant gene expression as gene knockdown had divergent effects on mean expression ratios in eu- versus hypoglycemic animals of each sex.

VMN aromatase function has a demonstrable impact on counterregulatory hormone secretion [24]; however, the phenotypic identity and distribution of VMN cells that express this critical enzyme remain unclear. Current observations that VMNdm Ghrh/SF-1 neurons contain aromatase mRNA infer that these cells are a likely local source of neuroestradiol. Co-expression of ER variant and aromatase mRNAs support the prospect that these receptors may transduce regulatory effects of extrinsic ligand of ovarian origin as well as autocrine/intrinsic estradiol signaling. Data here show that baseline aromatase gene transcription is subject to Ghrh-R inhibition in each sex, but transcription varies significantly between the two sexes as transcript levels in males exceed those in females. The prospect that VMNdm Ghrh/SF-1 nerve cell aromatase protein content and enzyme activity are corresponding lower in female versus male rats remains to be addressed. Data showing hypoglycemia-associated down-regulated aromatase gene expression in male, but not female VMNdm Ghrh/SF-1 neurons infer that neuroestradiol production may fall in response to declining plasma glucose levels in the former sex alone. Importantly, the reversal of this negative transcriptional response by Ghrh-R siRNA pretreatment infers that Ghrh-R signaling may be a primary effector of down-regulated ER ligand yield in male rat Ghrh/SF-1 neurons. For the male rat, it would be informative to learn how this likely change in neuroestradiol signal volume may affect ER regulation of these cells. A question of paramount interest concerns the total mass of neuroestradiol that is produced within this discrete nerve cell population and the approximate diffusion/concentration range of this steroid signal from the point of cellular origin. Further studies are needed to determine if neuroestradiol produced within VMNdm Ghrh/SF-1 neurons is adequate for sole activation of ERs expressed in the cell type or, on the other hand, is of sufficient magnitude to access ERs in neighboring cells.

In summary, present studies applied *in vivo* gene silencing and single-cell laser catapult microdissection/multiplex qPCR analytical tools to address the premise that Ghrh-R may regulate ER variant-specific estradiol input to VMNdm Ghrh/SF-1 neurons during eu- and/or hypoglycemia, according to sex. Results document glucose-dependent Ghrh-R control of VMNdm Ghrh/SF-1 neuron ER-alpha (female), ER-beta (both sexes), and GPER (both sexes) gene expression. Ongoing studies seek to characterize mechanisms that cause a hypoglycemia-associated gain of regulatory control or switch in direction (stimulatory-to-inhibitory) of control. Outcomes also point to VMNdm Ghrh/SF-1 as a local source of neuroestradiol in each sex and implicate Ghrh-R in hypoglycemic suppression of this neurosteroid profile in males. Estradiol acts on VMN targets to regulate systemic glucose homeostasis. Current data support the prospect that Ghrh-R may shape control of VMNdm Ghrh/SF-1 neurotransmission during glucose homeostasis or imbalance, in part, through regulation of ER variant-mediated estradiol input.

## Supplementary Material

Supplementary Figure
