# Supplementary material for: Sex-dimorphic growth hormone-releasing hormone (Ghrh) receptor regulation of ventromedial hypothalamic nucleus Ghrh neuron estrogen receptor variant gene expression
Source: Transl Neurosci. 2025 Jun 14;16(1):20250373. doi: 10.1515/tnsci-2025-0373 (PMC12176007; doi:10.1515/tnsci-2025-0373)
Supplement: Supplementary Figure [file tnsci-2025-0373-sm.pdf]

Supplementary material

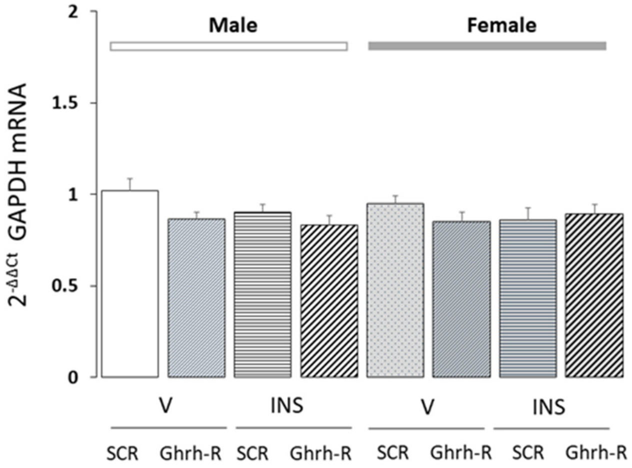

Figure S1: Groups of male and female rats (*n* = 6 males and *n* = 6 females per group) were pretreated by bilateral intra-VMN scramble (SCR) or growth hormone-releasing hormone receptor (Ghrh-R) siRNA administration seven days before subcutaneous (sc) injection of vehicle (V) or neutral protamine Hagedorn insulin (INS; 10.0 U/kg bw). Brain tissue was harvested by dissection one hour after injections. Individual Ghrh-immunopositive neurons were laser-catapult-microdissected from 10 micron-thick fresh frozen sections cut through the dorsomedial ventromedial hypothalamic nucleus (VMNdm). Data presented here depict mean normalized GAPDH mRNA measures + S.E.M. (depicted above each bar) for male (bars 1-4, at left) and female (bars 5-8, at right) rat treatment groups. Treatment groups are identified as follows: SCR siRNA/V (male: bar 1, *n* = 12; female: bar 4, *n* = 12); Ghrh-R siRNA/V (bar 2, *n* = 12; female: bar 6, *n* = 12); SCR siRNA/INS (male: bar 3, *n* = 12; female: bar 7, *n* = 12); Ghrh-R siRNA/INS (male: bar 4, *n* = 12; female: bar 8, *n* = 12). Gene transcript data were analyzed by three-way ANOVA and Student-Neuman-Keuls post-hoc test. Statistical analysis shows that GAPDH gene expression did not vary significantly among treatment groups.
